# Oxidized LDLs Inhibit TLR-induced IL-10 Production by Monocytes: A New Aspect of Pathogen-Accelerated Atherosclerosis

**DOI:** 10.1007/s10753-012-9472-3

**Published:** 2012-05-04

**Authors:** Małgorzata Bzowska, Anna Nogieć, Joanna Skrzeczyńska-Moncznik, Barbara Mickowska, Krzysztof Guzik, Juliusz Pryjma

**Affiliations:** 1 Department of Immunology, Faculty of Biochemistry, Biophysics, and Biotechnology, Jagiellonian University, Gronostajowa 7, 30-387 Kraków, Poland; 2 Malopolska Centre of Food Monitoring and Certification, Faculty of Food Technology, Agricultural University, Balicka 122, 30-149 Kraków, Poland

**Keywords:** IL-10, TLR, monocytes, lipoproteins, atherosclerosis

## Abstract

It is widely accepted that oxidized low-density lipoproteins and local infections or endotoxins in circulation contribute to chronic inflammatory process at all stages of atherosclerosis. The hallmark cells of atherosclerotic lesions—monocytes and macrophages—are able to detect and integrate complex signals derived from lipoproteins and pathogens, and respond with a spectrum of immunoregulatory cytokines. In this study, we show strong inhibitory effect of oxLDLs on anti-inflammatory interleukin-10 production by monocytes responding to TLR2 and TLR4 ligands. In contrast, pro-inflammatory tumor necrosis factor secretion was even slightly increased, when stimulated with lipopolysaccharide from *Porphyromonas gingivalis—*an oral pathogen associated with atherosclerosis. The oxLDLs modulatory activity may be explained by altered recognition of pathogen-associated molecular patterns, which involves serum proteins, particularly vitronectin. We also suggest an interaction between vitronectin receptor, CD11b, and TLR2. The presented data support a novel pathway for pathogen-accelerated atherosclerosis, which relies on oxidized low-density lipoprotein-mediated modulation of anti-inflammatory response to TLR ligands.

**Electronic supplementary material:**

The online version of this article (doi:10.1007/s10753-012-9472-3) contains supplementary material, which is available to authorized users.

## INTRODUCTION

Atherosclerotic vascular disease remains the most common cause of death and disability in westernized countries. Although atherosclerosis was recognized for many years as a simple lipid storage disease, nowadays, it is widely accepted that both—disturbed lipid metabolism leading to accumulation of low-density lipoproteins (LDLs) in the arteries and chronic inflammation of the vascular wall—are the most prominent features of this illness [1, 2]. Even though, oxidized forms of low-density lipoproteins (oxLDLs) are major risk factor, recently accumulating evidences have implicated that infectious agents can accelerate atherosclerosis [3–5]. It has been documented that certain chronic infections such as periodontitis and chlamydial infection exacerbate clinical manifestation of atherosclerosis [6, 7]. The presence of the pathogen-associated molecular patterns (PAMPs), like lipopolysaccharide (LPS), peptidoglican, and bacterial DNA, which are shed and released by all growing and dividing bacteria, was demonstrated in significant proportion of lesions. It is believed that predominantly PAMPs and only exceptionally viable microbes accumulate within atheroma [8–10]. Thus, human atherosclerosis lesions may be directly exposed to bacterial ligands of Toll-like receptors (TLR). TLR receptors is a family of innate immune recognition receptors that detect PAMPs and play a key role in initiating inflammatory responses and, most likely, in the pathogenesis of atherosclerosis [11–14]. TLRs are expressed on monocytes and monocyte-derived macrophages, which are involved in the atherosclerotic lesion development as well as in coordination of innate and acquired immune responses. Cells of monocytic lineage express a variety of receptors, which enable them to detect and integrate complex signals derived from other cells, lipoprotein products, and pathogens (reviewed in [15]). As immunoregulatory cells, in response to stimuli, monocytes and macrophages produce considerable amounts of pro- and anti-inflammatory cytokines, which in the context of atherosclerosis can be considered as pro- or anti-atherogenic (reviewed in [16]) and play an important role in the development, progression, and complications of atherosclerosis.

Tumor necrosis factor (TNF) as a potent proinflammatory cytokine is involved in the induction of expression of adhesion molecules and chemokines in the vascular wall, the first step during development of atherosclerotic lesions. Its key role in atherosclerosis was demonstrated in mice model showing that atherosclerotic lesion size was significantly smaller in animals deficient in TNF, which was associated with decreased expression of intracellular adhesion molecule-1 (ICAM-1), vascular cell adhesion molecule-1 (VCAM-1), and monocyte chemotactic protein-1 (MCP-1) [17]. Moreover, it was shown that TNF level correlates strongly with the burden of atherosclerosis in healthy middle-aged men, severity of peripheral arterial disease, and also elevated risk of recurrent myocardial infarction [18]. In contrast, interleukin-10 (IL-10) is a powerful antiatherogenic cytokine. As a pleiotropic, anti-inflammatory cytokine, it inhibits a broad array of immune functions. The role of IL-10 has been clearly established in mouse model of atherosclerosis. It was demonstrated that IL-10 deficiency promotes early atherosclerotic lesions formation [19]. Consistent with a protective role of IL-10 in atherosclerosis, overexpression of IL-10 decreases formation of early fatty streak, prevents exaggerated advanced atherosclerosis development, and modulates cellular and collagen plaque composition in mice [20–22]. Furthermore, elevated IL-10 serum levels were associated consistently with significantly improved endothelial function and outcome of patients with acute coronary syndrome [23, 24]. It is believed that the balance between pro- and anti-inflammatory factors and magnitude of cytokine release at a site of lesion formation may affect further disease fate. In early stages of atherosclerosis, cytokine balance can alter endothelial function favoring or hampering the recruitment, adherence, and migration of leucocytes into the inflamed vessel wall. At a more advanced stage of disease, pro- and anti-inflammatory balance may regulate processes of atherosclerotic plaques destabilization. For example, the imbalance between matrix degradation and synthesis, destroying fibrous cap structure and leading to its rupture was correlated with prevailing proinflammtory cytokine expression over IL-10 and transforming growth factor secretion (reviewed in [16]). In the context of atherosclerosis, research has focused on the potential of proatherogenic lipids to modulate proinflammatory events, but as atherosclerosis is a chronic inflammatory disease, regulation of anti-inflammatory cytokines may be equally if not more important. As a matter of fact, experimental and epidemiological data support the concept that endothelial function, plaque instability, and patient outcome in atherosclerotic vascular disease depend on the pro- and anti-inflammatory balance at the sites of disease development.

What has hitherto remained unexplained is if oxLDLs influence cytokine production activated by innate recognition of PAMPs, if such regulation favors pro- or anti-inflammatory response, and if other serum factors, which are not considered to interact directly with TLRs, participate in this regulation. Therefore, we set out to challenge these concepts in a simple experimental model employing human peripheral blood monocytes or derived macrophages, a set of well-defined TLR ligands and readily reproducible oxLDLs. We suppose that this model may reflect the pathophysiological *milieu* during development of atherosclerosis lesions when monocytes are exposed directly to proatherogenic lipids and pathogen-associated molecules.

## MATERIALS AND METHODS

### Human Peripheral Blood Monocytes and Monocyte-Derived Macrophages

Peripheral blood mononuclear cells (PBMCs) were isolated from citrate-treated blood of healthy donors using standard density gradient centrifugation (Ficoll-Paque PLUS, Amersham Biosciences, Uppsala, Sweden) and plated at 3.5 × 10^6^/well in 24-well Cell + plates (Sarstedt, Newton, NC, USA) in RPMI1640 (Gibco Invitrogen Corp., Paisley, UK) supplemented with 2 mM l-glutamine, 50 μg/ml gentamycin (Sigma), in later parts of text called complete medium, and 10 % fetal calf serum (FCS, Biochrom). After 2 h of incubation at 37°C in humidified atmosphere containing 5 % CO_2_, nonadherent cells were removed by washing with complete medium. To obtain elutriation-purified nonadherent monocytes, PBMCs were subjected to counterflow centrifugation as described previously [25]. The monocytes phenotype was routinely controlled (in the case of adherent cells after nonenzymatic detachment) by immunofluorescence staining with mAb anti-CD14 (clone: TŰK4, DakoCytomation) and subsequent flow cytometry analysis (LSRII, Becton Dickinson). The cultures selected for further experiments were positive in at least 90 % for the CD14. To obtain monocyte-derived macrophages, adherent monocytes were cultured in complete medium supplemented with 10 % pooled heat-inactivated human serum (HS) for at least 7 days. The medium was changed every 2 days. The human monocytes-derived macrophages (hMDMs) phenotype was routinely controlled, after nonenzymatic detachment of cells, by immunofluorescence staining of CD14 (clone: TŰK4, DakoCytomation), CD11b (clone: ICRF44, Becton Dickinson and Co, Franklin Lakes, USA), and CD209 (clone: DCN46, Becton Dickinson) and subsequent flow cytometry analysis. The cultures selected for further experiments were positive in at least 90 % for the first two markers and <1 % for CD209. The adherent cells acquired typical macrophage morphology. Resting (unstimulated) cells did not produce cytokines: IL-1, TNF, IL-6, and IL-10.

### Isolation and Oxidation of Lipoproteins

Low density lipoproteins (LDLs) were isolated from the fresh EDTA-treated plasma of healthy donors by the method of sequential ultracentrifugation through a discontinuous KBr gradient according to Havel *et al*. [26]. Plasma density was adjusted to 1.019 g/ml with solid KBr, and plasma was centrifuged at 180,000×*g* for 24 h at 4°C (Beckman L7-65 ultracentrifuge with Ti 60 rotor, Beckman, USA). The top fraction containing very low-density lipoproteins and intermediate density lipoproteins was removed and density of the remaining solution was raised to 1.063 g/ml by addition of solid KBr followed by repeated centrifugation. LDL fraction from the top layer was collected and dialysed overnight at 4°C against phosphate-buffered saline (PBS) with 0.05 % EDTA pH 7.4 for native LDLs (nLDLs) or against PBS pH 7.4 for LDLs prepared to be oxidized. Oxidation of LDLs was performed by incubation with Cu^2+^ for 20 h at 37°C (final Cu^2+^ concentration, 5 μM) after adjustment of protein concentration to 0.12 mg/ml. Obtained oxLDLs were dialyzed overnight against PBS pH 7.4 at 4°C. nLDLs and oxLDLs preparations were concentrated by ultrafiltration (Amicon Ultra Centifugal Filters,100 K NMWL, Millipore, USA) at 3,500×*g* at 4°C, and sterilized by filtration through a 0.22-μm syringe filter (Millex-GV, Millipore, USA). Minimally modified LDLs were obtained by storage of nLDLs at 4°C for 6 months. All materials used during isolation procedures were endotoxin free, and we have not observed LDL-induced stimulation of cytokine production. Protein concentration was quantified by the Lowry method using Total Protein Kit (MicroLowry, Peterson’s Modification, Sigma, USA). The purity of LDLs preparations was routinely controlled by polyacrylamide gel electrophoresis and subsequent gels staining with Red Oil O for lipids detection (Sigma, USA) and with Coomasie Briliant Blue R for protein detection (Sigma, USA).

### Treatment with LDLs and Stimulation

In most experiments elutriation-purified, adherent monocytes or macrophages were placed in complete medium supplemented with 10% FCS, treated for 30 min with native, minimally modified, or oxidized low-density lipoproteins (0–50 μg/ml) and then stimulated with PAMPs: *Escherichia coli* 0127:B8 LPS (stLPS, Sigma), ultrapure *E. coli* 011:B4 LPS (upLPS, Invivogen), synthetic lipopeptides: diacylated Pam2CysSerLys4 (Pam2, Invivogen), and triacylated Pam3CysSerLys4 (Pam3, Invivogen) at a final concentration of 10 ng/ml, ultrapure *Porphyromonas gingivalis* LPS (pgLPS, Invivogen) at a final concentration of 1 μg/ml. In some cases, cells were pretreated with oxidized LDLs as described above or in complete medium supplemented with 0.15 % bovine serum albumin (BSA, Sigma). After pretreatment, oxLDLs were washed out, and cells were stimulated in complete medium supplemented with 10 % FCS. For the experiments concerning the influence of soluble serum factors on cytokine production, elutriation-purified monocytes were placed in culture plates or in polypropylene culture tubes (BD Falcon) in complete medium supplemented with 0.15 % BSA, 1 %, 10 %, 30 % FCS, or 10 % HS and stimulated as described above.

### Treatment with LDLs and Immunofluorescence Staining

To analyze the effect of oxLDLs on expression of surface receptors on monocytes, cells were placed in complete medium supplemented with 10 % FCS and treated for 30 min or 3 h with oxidized low-density lipoproteins (15 μg/ml). After nonenzymatic detachment monocytes were suspended in complete medium supplemented with 5% FCS (5 × 10^5^/sample/100 μl) and incubated with PE-conjugated antihuman TLR2 (clone TL2.1, eBiosciences), TLR4 (clone HTA125, eBiosciences), CD36 (clone CB38, BD Pharmingen), CD11b (clone ICRF44, BD Pharmingen) or Fluorescein isothiocyanate (FITC)-conjugated antihuman CD14 (clone RM052, Immunotech) mAbs, or appropriate isotype controls (BD Pharmingen) for 30 min at 4°C. After washing with cold medium, the cells were resuspended and analyzed by flow cytometry using an LSRII cytometer (Becton Dickinson). The analysis was performed using the FACSDiva program to determine the percentage and mean fluorescence intensity (MFI) of positive cells.

### Blocking of TLR Receptors

Adherent monocytes were placed in complete medium supplemented with 10% FCS and incubated with 10 μg/ml of blocking monoclonal antibodies against human TLR2 (clone: TL2.1, IgG2a, eBiosciences) or TLR4 (clone: HTA125, IgG2a, eBiosciences) or appropriate isotype controls (eBiosciences) for 30 min at room temperature (RT). Then, the cells were stimulated with LPS from *P. gingivalis* at final concentration 1 μg/ml for 20 h, and supernatants were collected.

### Regulation of αM, αvβ3, and αvβ5 Integrins

Adherent monocytes were placed in complete medium supplemented with 1% FCS and incubated with ligands for integrins: recombinant human ICAM-1 (10 μg/ml, R&D), human plasma fibrinogen (20–200 μg/ml, Millipore), fibronectin (0.1–1 μg/ml, Millipore), or vitronectin (3–30 μg/ml, Millipore) for 30 min at 37°C in humidified atmosphere containing 5 % CO_2_. Alternatively, cells were placed in complete medium supplemented with 10 % FCS and incubated with 150 mM *N*-acetyl-d-glucosamine (Sigma), 10 μg/ml of blocking monoclonal antibodies against human CD11b (αM integrin, clone Vim12, IgG1, Santa Cruz Biotechnology, Inc.) or agonistic monoclonal antibodies against human β3 (clone RUU-PL 7 F12, IgG1, BD Pharmingen), αvβ3 (clone LM609, IgG1, Chemicon), and αvβ5 (clone P1F6, IgG1, Chemicon) integrins for 30 min at RT. For applied antibodies corresponding isotype controls (BD Pharmingen) were used, all at concentration 10 μg/ml. Then, the cells were stimulated with LPS from *P. gingivalis* at final concentration of 1 μg/ml for 20 h, and supernatants were collected.

### Membrane Disruption

Membrane-disrupting agent methyl-β-cyclodextrin (MβCD, Sigma) was added to monocytes at 0.5 and 1 mM. Then, the cells were stimulated with LPS from *E. coli* or *P. gingivalis* at final concentration 10 ng/ml or 1 μg/ml for 20 h. Then, supernatants were collected, and cell viability was monitored by propidium iodide exclusion test.

### Viability Assay

Cells were evaluated for viability and apoptosis after LDLs treatment by morphology using bright-field microscopy or by the binding of FITC-labeled annexin V and exclusion of propidium iodide (PI) according to the manufacturer’s recommendations (Annexin V-FITC kit, Bender MedSystem, Vienna, Austria) followed by analysis with LSR II flow cytometer (Becton Dickinson).

### Measurement of IL-10 and TNF Production

For the cytokine measurements, supernatants were collected at different time points after stimulation as indicated in the figures. All supernatants were centrifuged at 500×*g* for 5 min to remove particulate debris and stored at −20°C. The concentrations of IL-10 and TNF in culture supernatants were determined by enzyme-linked immunosorbent assays (ELISAs) using the OptEIA Sets (BD Pharmingen) according to the instructions provided with each set of antibodies. The assay was sensitive down to concentration of 7 pg/ml. Selected data (Fig. 2c, d) were presented as IL-10/TNF biological activity ratio obtained from recalculation of ELISA results to international units of biological activity per ml (based on data of ELISA standards against NIBSC/WHO international standards).

### Statistical Analyses

All experiments were performed at least in triplicate. The data are presented as means ± SD. All statistics were calculated using Origin 8.1 (OriginLab Corporation, Northampton, MA, USA). Statistical significance was asset at 0.05 and calculated using one-way ANOVA test.

## RESULTS

### OxLDLs Inhibit TLR2- and TLR4-Dependent Production of IL-10

**Fig. 1:**
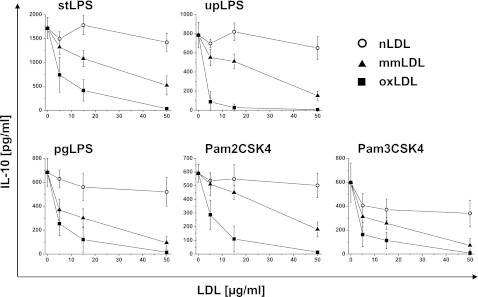
Oxidized LDLs inhibit TLR2- and TLR4-dependent production of IL-10. Monocytes were isolated from PBMC by adherence and placed in media supplemented with 10 % FCS. Cells were treated for 30 min with native (nLDLs; *open circles*), minimally modified (mmLDLs; *black triangles*) or oxidized (oxLDLs; *black squares*) low-density lipoproteins at the indicated concentrations and then stimulated with *E. coli* LPS (stLPS and upLPS), *P. gingivalis* LPS (pgLPS), Pam2CSK4 or Pam3CSK4. Supernatants were collected 20 h after stimulation and IL-10 concentrations were determined by ELISA. Data presented are mean ± SD from at least three independent experiments.

The first series of experiments was aimed at investigating the ability of oxLDLs to modulate anti-inflammatory cytokine IL-10 production by monocytes. Cells were isolated from PBMC by adherence or elutriation-purified, and treated with native (nLDLs), minimally modified (mmLDLs), or oxLDLs for 30 min. As control, untreated cells were maintained in culture for the same time. Monocytes were stimulated with defined PAMPs specific for TLR4, TLR2, or its heterodimers with TLR1 or TLR6. Supernatants were collected after 20 h, and IL-10 concentrations were measured. Unstimulated cells did not secrete IL-10, and oxLDLs alone did not activate cytokine production as well (data not shown). As shown in Fig. 1, both oxidized and minimally modified LDLs suppressed the TLR dependent production of IL-10 by monocytes in a dose-dependent manner. Response to challenge with stLPS, pgLPS, and Pam2CSK4 was inhibited by oxLDLs in about 50 % at concentration 5 μg/ml and reached about 80 % at concentration 15 μg/ml. IL-10 production induced by upLPS and Pam3CSK4 was inhibited by oxLDLs by about 80 % already at concentration 5 μg/ml. Moreover, this inhibition depended on LDLs oxidation state as native LDLs did not alter the production of IL-10 (Fig. 1). For Pam3CSK4 stimulation, we observed slight inhibition by nLDLs, but still, reduction of IL-10 production was much stronger in the presence of oxidized LDLs. The observed effect was specific for oxLDLs as high-density lipoproteins (HDLs) did not influence the IL-10 secretion even oxidated (data not shown). The inhibitory effects were not attributable to lipoprotein toxicity as we carefully studied the viability of monocytes incubated with native, minimally modified and oxidized LDLs at concentrations from 1 to 100 μg/ml. As clearly indicated by staining with annexin V and propidium iodide exclusion test, the presence of LDLs had no effect on monocyte viability (data not shown). In summary, our data consistently demonstrate that oxLDLs inhibited TLR2- and TLR4-dependent IL-10 production by monocytes.

### Effect of Oxidized LDLs on PAMPs-Induced IL-10 and TNF Production

**Fig. 2:**
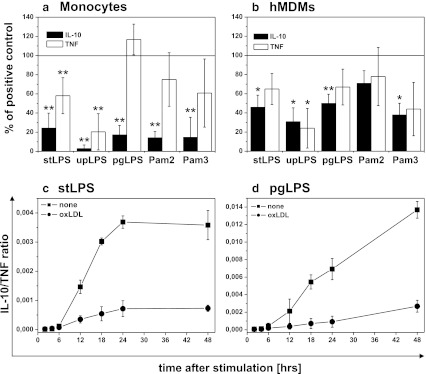
Effect of oxidized LDLs on PAMPs-induced IL-10 and TNF production in monocytes and monocyte-derived macrophages (hMDM). **a**, **b** Monocytes were isolated from PBMC by adherence and placed in media supplemented with 10 % FCS. hMDMs were differentiated from adherent monocytes for at least 7 days in medium supplemented with 10 % human serum, and before experiment, cells were placed in media supplemented with 10 % FCS. Cells were cultured alone or treated for 30 min with oxidized LDLs at the 15 μg/ml and then stimulated with PAMPs as indicated in the *X*-axis. Supernatants were collected 20 h after stimulation and IL-10 (*black bars*) and TNF (*white bars*) concentrations were determined by ELISA. Data are expressed as percent of positive control that is cells stimulated in the absence of oxLDLs (indicated as 100 %). Values are the mean ± SD from at least five independent experiments. **p < 0.01, *p < 0.05 *versus* corresponding positive controls. Absolute cytokine levels are presented in supplementary data Table 1. **c**, **d** Monocytes were isolated from PBMC by adherence and placed in media supplemented with 10 % FCS. Cells were cultured alone (*black squares*) or treated for 30 min with oxidized LDLs at the 15 μg/ml (*black circles*), and then stimulated with stLPS (**c**) or pgLPS (**d**). Supernatants were collected at time points indicated in the figure, and IL-10 and TNF concentrations were determined by ELISA. For each time point, IL-10/TNF biological activity ratios recalculated as described in “Materials and Methods” are presented in the figure (**c**, **d**). Values are the mean ± SD from three independent experiments. Absolute cytokine levels are presented in supplementary data Figure 1. Similar experiments were done with upLPS, Pam2CSK4, or Pam3CSK4 (data not shown).

In order to determine whether oxLDLs change the pro- and anti-inflammatory balance, we concurrently measured the production of IL-10 and TNF by monocytes and hMDMs. The data presented in Fig. 2a and supplementary data Table 1 show that the ability of oxLDLs to modulate TNF production by monocytes depended on ligand used to induce the cytokine secretion. Although for upLPS stimulation TNF production was strongly inhibited, secretion of TNF induced with stLPS, Pam2CSK4, and Pam3CSK4 was only moderately reduced. Surprisingly, oxLDLs did not suppress TNF production stimulated by pgLPS—while IL-10 production was inhibited, TNF secretion was even slightly increased. At the same time, the effect of oxLDLs on TLR-dependent cytokine production by hMDMs was completely different (Fig. 2(b) and supplementary data Table 1), namely, the secretion of both cytokines, was similarly suppressed.

Consequently, we have also done kinetics experiments and determined cytokine concentrations in supernatants at distinct time points after stimulation (supplementary data Figure 1). We have observed that TNF was secreted very quickly (maximum at 6–18 h), and subsequently, the concentration of TNF decreased. The TNF decline was concomitant with increased production of IL-10 (maximum at 24 h). Results presented in Fig. 2a and b were obtained from supernatants collected 20 h after stimulation, that is at maximum for IL-10 secretion. Based on kinetics experiments, we excluded the possibility that oxLDL-mediated modulation of cytokine production resulted from altered kinetics of cytokine release (supplemetary data Figure 1). We recalculated our results as IL-10/TNF biological activity ratio at distinct time points in cultures with or without oxLDLs as described in “Materials and Methods.” As shown in Fig. 2c and d, after initial intensive TNF production, the anti-inflammatory reaction started as indicated by considerable increase IL-10/TNF ratio (squares). While for TLR4 ligands, the ratio reached plateau at 24 h; for TLR2 ligands, it was growing up constantly for 48 h, mainly due to much more rapid drop in TNF concentration in supernatants. What is more important, irrespectively of oxLDLs-mediated inhibitory effect on both cytokines or only IL-10 production, IL-10/TNF ratios at distinct time points were much lower in the presence of oxLDLs (Fig. 2c and d circles). The results allow to conclude that oxLDLs hindered expansion of anti-inflammatory response following less or more intense pro-inflammatory reaction.

### Oxidized LDLs Do Not Affect Surface Expression of TLR2, TLR4 and Their Coreceptors CD14, CD11b, and CD36

**Fig. 3:**
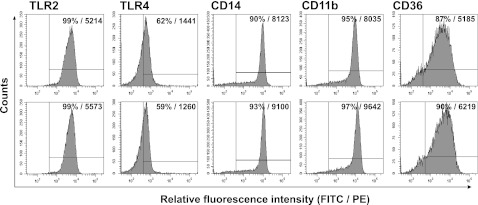
Oxidized LDLs do not affect surface expression of TLR2, TLR4, and their coreceptors CD14, CD11b, and CD36. Control (*upper panel*) or treated for 3 h with oxLDLs (*lower panel*) monocytes were stained with PE-conjugated antihuman TLR2, TLR4, CD36 mAbs, or FITC-conjugated antihuman CD14, CD11b mAbs and analyzed by flow cytometry. The histogram markers were set based on the signal from appropriate isotype controls, and they designate section of the histogram representing positive cells. The percent of positive cells and MFI values are given in histograms. Results of a representative experiment of three performed are presented.

As we observed oxLDL-mediated inhibition of IL-10 production by monocytes stimulated with TLR ligands, we studied whether oxLDLs affect expression of TLR2, TLR4, and their coreceptors on monocytes. We have measured binding of TLR2-, TLR4-, CD14-, CD11b-, and CD36-specific mAbs to cells treated with oxLDL for 30 min (data not shown) and 3 h (Fig. 3). Results presented in Fig. 3 clearly demonstrate that oxLDLs did not decrease surface expression of tested receptors, which was also apparent after short, 30 min treatment. Contrary, we noticed slight increase in fluorescence intensities for TLR2, CD14, CD11b, and CD36. Consequently, we assumed that diminished production of IL-10 secretion following treatment with oxLDLs cannot be attributed to reduced expression of TLRs and/or their coreceptors.

### IL-10 and TNF Production Depends on Serum Soluble Factors

**Fig. 4:**
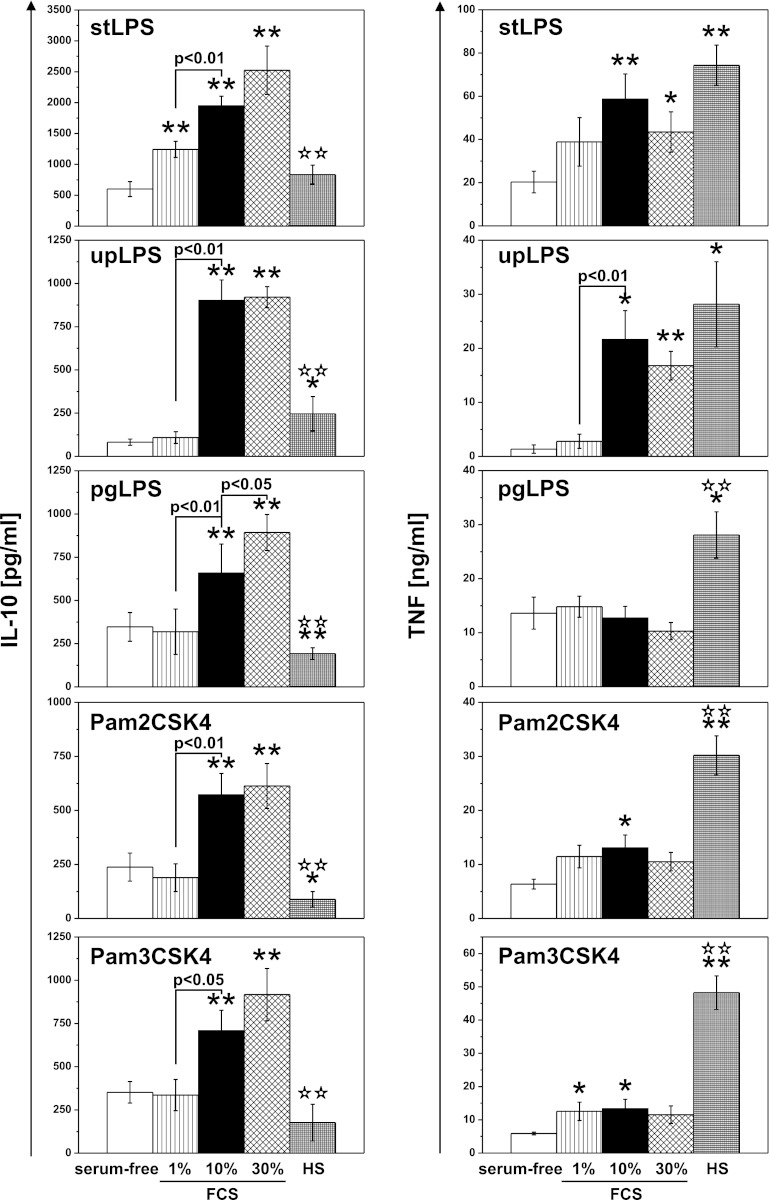
IL-10 and TNF production depends on serum soluble factors. Monocytes were isolated from PBMC by adherence. Before stimulation cells were placed in media supplemented with 0.15 % BSA (serum-free; *white bars*) or 1 % (*dashed bars*), 10 % (*black bars*), 30 % (*checked bars*) FCS or 10 % HS (*dense-checked bars*), and then activated with PAMPs: *E. coli* LSP (stLPS and upLPS), *P. gingivalis* LPS (pgLPS), Pam2CSK4, or Pam3CSK4. Supernatants were collected 20 h after stimulation and IL-10 (*left panel*) and TNF (*right panel*) concentrations were determined by ELISA. Values are the mean ± SD from three independent experiments. **p < 0.01 and *p < 0.05 *versus* serum-free conditions, **p < 0.01 *versus* 10 % FCS.

**Fig. 5:**
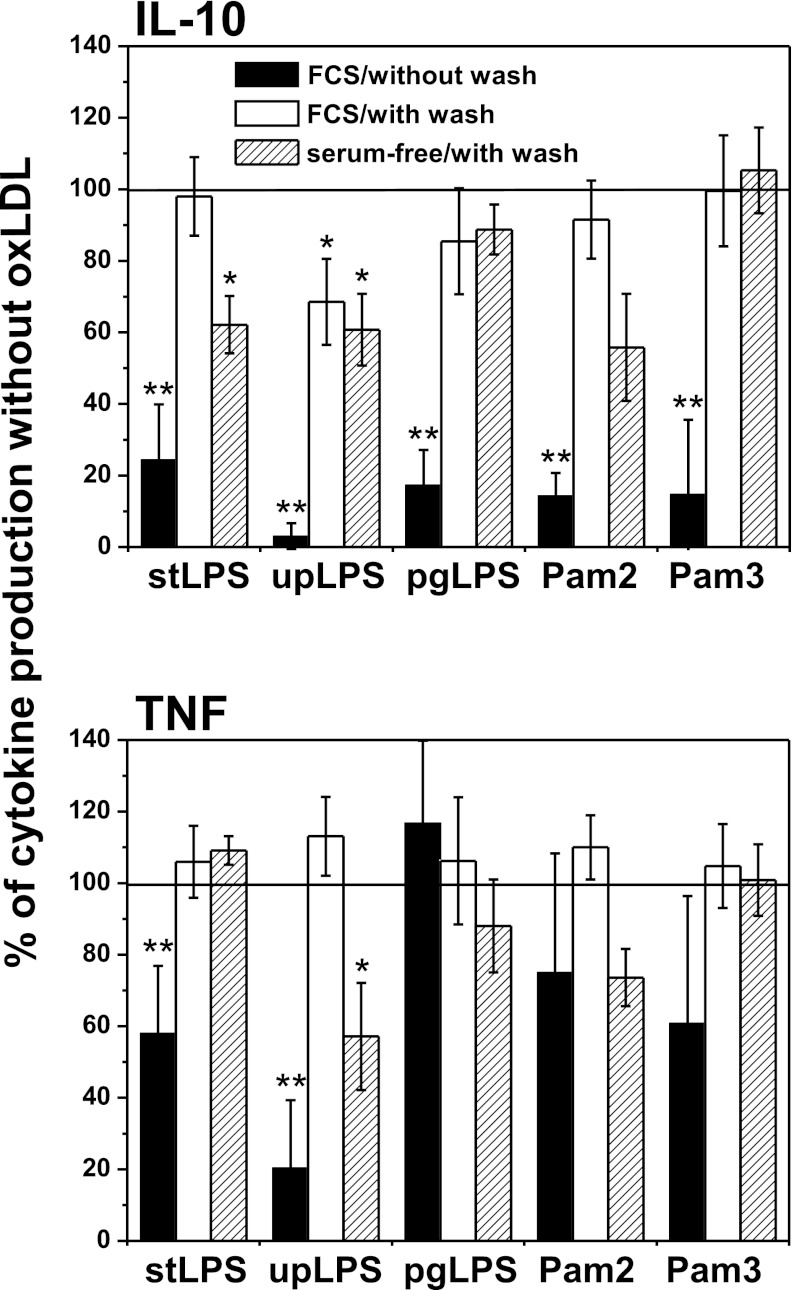
Oxidized LDLs inhibit both extracellular and cell-associated components of TLR2 and TL4 activation. Monocytes were isolated from PBMC by adherence and placed in media supplemented with 10 % FCS. Cells were cultured alone or treated for 30 min with oxidized LDLs at the 15 μg/ml, and then stimulated (*black bars*) or pretreated with oxLDLs, washed, and stimulated (*white bars*) with PAMPs: *E. coli* LSP (stLPS and upLPS), *P. gingivalis* LPS (pgLPS), Pam2CSK4, or Pam3CSK4. In parallel, cells were pretreated for 30 min with oxLDLs in serum-free medium, washed, and stimulated with PAMPs (*dashed bars*) in media supplemented with 10 % FCS. Supernatants were collected 20 h after stimulation and IL-10 (*top*) and TNF (*bottom*) concentrations were determined by ELISA. Data are expressed as percent of positive control that is cells stimulated in the absence of oxLDLs (indicated as 100 %). Values are the mean ± SD from three independent experiments, **p < 0.01, *p < 0.05 *versus* corresponding positive controls.

It was shown that the inhibitory effect of oxidized phospholipids (oxPLs) was in large part attributable to interaction with accessory molecules, namely, LPS-binding protein (LBP) and soluble CD14 (sCD14), participating in TLR activation [27, 28]. Therefore, we assumed that serum soluble molecules also may take part in the described regulation of IL-10 and TNF secretion. To answer this question, we stimulated monocytes with TLR ligands in serum-free medium or in media supplemented with 1 %, 10 %, and 30 % FCS, or 10 % HS. As shown in Fig. 4, production of both cytokines was equally dependent on FCS when stimulated with LPS from *E. coli*. In serum-free media and at 1 % of FCS, upLPS-induced production of IL-10 as well as TNF was minimal and reached the maximum at 10 % of FCS. In monocytes stimulated with stLPS, we observed production even in the absence of FCS but alongside with raising concentration of FCS in media secretion of IL-10 and TNF increased, reaching the maximum for TNF at 10 % and for IL-10 at 30 % of FCS. Interestingly, in milieu with human serum, TLR2- and TLR4-induced IL-10 secretion was diminished, and TNF production was augmented in comparison to the cell cultures with appropriate concentration of FCS. Surprisingly, for TLR2-induced production of IL-10 and TNF, we observed distinct dependence on FCS concentration. All three applied TLR2 ligands induced similar IL-10 secretion in serum-free media and at 1 % FCS, and IL-10 production was strongly augmented in media supplemented with 10 % FCS. Interestingly, for maximal TNF production, 1 % of FCS was sufficient, and further rising of FCS concentration did not take effect or even suppressed TNF secretion. Noteworthy, the pgLPS-induced TNF production seemed completely independent on serum. On the basis of obtained results, we suggest that distinct effect of oxLDLs on TLR-induced production of IL-10 and TNF resulted from different dependence on soluble factors, which are main targets of oxLDLs inhibitory activity. To extend our observation, we had also preincubated monocytes with oxLDLs, then washed them with fresh medium and stimulated with PAMPs in the absence of oxLDLs. The removal of oxLDLs before stimulation led to complete reconstitution of TNF production induced by all used PAMPs. IL-10 secretion was also restored, with the exception of upLPS stimulated, where we observed significant but incomplete reversal of oxLDLs inhibitory effect (Fig. 5, white bars). It was suggested by other authors that oxPLs inhibit also cell-associated steps in PAMPs recognition and signaling [27, 28]. Supposing that affinity of oxLDLs to serum soluble and cellular factors differs, it cannot be excluded that cells preincubation with oxLDLs in medium with FCS mask interaction with cell-associated molecules. Therefore, we preincubated the cells with oxLDLs in serum-free medium, then washed them out and stimulated in the presence of 10 % FCS (Fig. 5, dashed bars). We observed complete restoration of Pam3CSK4- and pgLPS-induced IL-10 production. For the other stimulators, inhibition was still noticeable. Interestingly, the increased production of pgLPS-stimulated TNF disappeared after washing out of oxLDLs, especially when preincubated without serum.

### PgLPS-Induced IL-10 Production is Entirely Dependent on TLR2

**Fig. 6:**
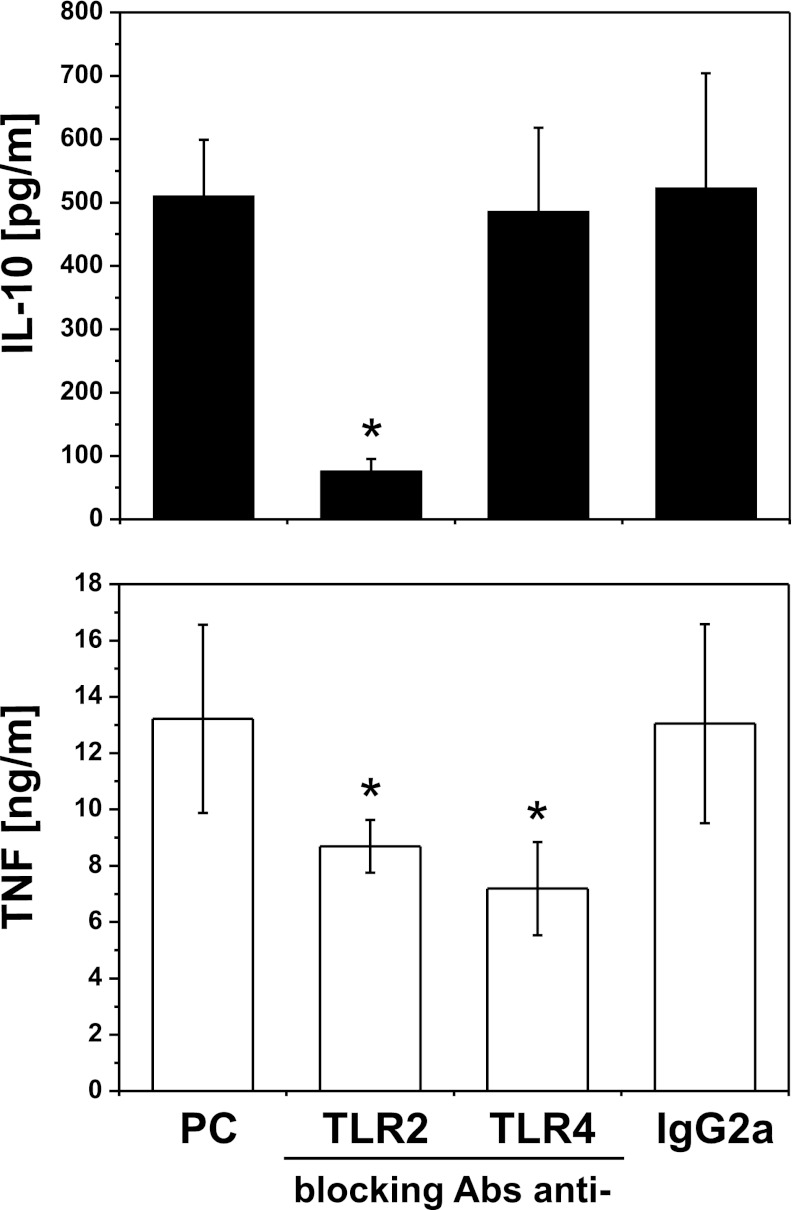
PgLPS-induced IL-10 production is entirely dependent on TLR2. Monocytes were isolated from PBMC by adherence and placed in media supplemented with 10 % FCS. Cells were culture alone (positive control, PC) or were pretreated for 30 min with blocking antibodies anti-TLR2, TLR4, or appropriate isotype control (IgG2a) at a concentration of 10 μg/ml, and stimulated with LPS from *P. gingivalis*. Supernatants were collected 20 h after stimulation, and IL-10 (*black bars*) and TNF (*white bars*) concentrations were determined by ELISA. Values are the mean ± SD from three independent experiments. Blocking and control antibodies alone did not induce cytokine secretion (data not shown). *p < 0.05 *versus* positive control that is cells stimulated with pgLPS in the absence of antibodies.

Recognition of LPS from *P. gingivalis* is still controversial. It was demonstrated to be an agonist for TLR2 and TLR4 and also an antagonist for TLR4 [29–31]. Such contradictory data could result, from different preparations of LPS, distinct cell models, and readout molecules employed to measure cell activation. To determine the contribution of TLR to pgLPS-induced production of IL-10 and TNF in monocytes, we used neutralizing antibodies to block TLR2 or TLR4. We have shown that proper isotype control did not influence cytokine production. To our surprise, we observed that IL-10 production in response to pgLPS was exclusively TLR2 dependent, while TNF secretion was inhibited partially by both antibodies (Fig. 6).

### TLR2-Dependent IL-10 Production is Regulated by Integrins

**Fig. 7:**
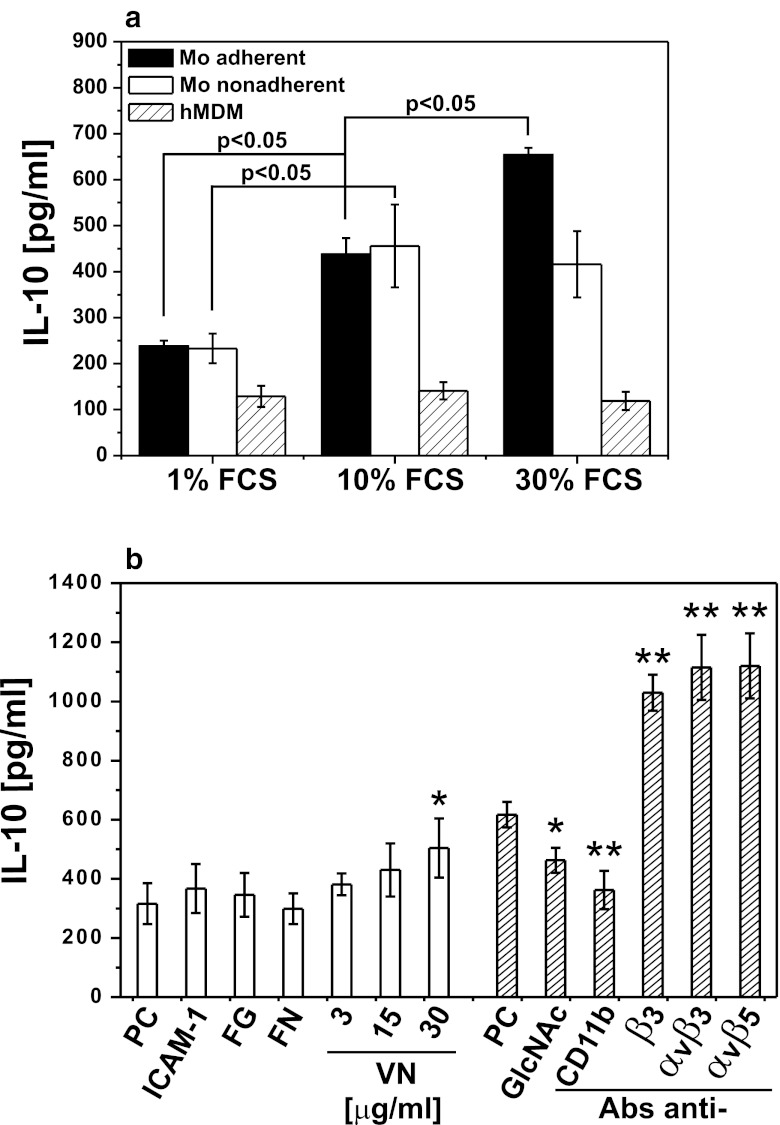
Integrins αM, αvβ3, and αvβ5 influence TLR-2-dependent IL-10 production. **a** Monocytes were isolated from PBMC by elutriation and placed in culture plates (Mo adherent, *black bars*) or in polypropylene culture tubes (Mo nonadherent, *white bars*). hMDMs were differentiated from adherent monocytes for at least 7 days in medium supplemented with 10 % human serum. Before experiments, cells were placed in media supplemented with 1 %, 10 %, or 30 % FCS and stimulated with pgLPS. Supernatants were collected 20 h after stimulation, and IL-10 concentrations were determined by ELISA. Values are the mean ± SD from three independent experiments; *p* values were determined for the different cell culture conditions. **b** Monocytes were isolated from PBMC by adherence and placed in media supplemented with 1 % FCS (*white bars*). Cells were cultured alone or pretreated for 30 min with: intercellular adhesion molecule 1 (ICAM-1; 10 μg/ml), fibrinogen (FG; 100 μg/ml), fibronectin (FN; 1 μg/ml), or vitronectin (VN; 3–30 μg/ml). Alternatively, cell were placed in media supplemented with 10 % FCS (*dashed bars*) and cultured alone or pretreated for 30 min with: *N*-acetyl-d-glucosamine (GlcNAc, 150 mM), blocking mAb against αM integrin (CD11b, clone Vim12) or agonistic mAbs against β3 integrin (clone RUU-PL 7F12), αvβ3 (clone LM609), αvβ5 (clone P1F6), all at a concentration 10 μg/ml. Then, cells were stimulated with LPS from *P. gingivalis*. Supernatants were collected 20 h after stimulation, and IL-10 concentrations were determined by ELISA. In the case of blocking or agonistic mAbs, the corresponding isotype controls were without effect and were not included in the figure. Values are the mean ± SD from three independent experiments. PC indicates positive control that is cells cultured in 1 % FCS or 10 % FCS medium and stimulated with pgLPS. **p < 0.01, *p < 0.05 *versus* corresponding positive controls.

Because of specific IL-10-promoting effect of serum, we searched for some still unrecognized serum “accessory molecules.” From the results demonstrated in Fig. 7a, we inferred that the serum effect was evident only in freshly adhered monocytes (black bars) in contrast to cells with established adherence—hMDM (dashed bars) or monocytes—which adherence was denied by incubation in polypropylene tubes—nonadherent monocytes (white bars). We concluded that the serum “accessory molecule” could be one of its adherence factor, and consequently, we measured IL-10 production by adherent monocytes stimulated with pgLPS in media supplemented with purified fibronectin (FN) or vitronectin (VN). We observed concentration-dependent augmentation of IL-10 secretion when vitronectin substituted for FCS. Additionally, pgLPS-stimulated IL-10 production was increased as a result of costimulation of β3, αvβ3, and αvβ5 integrins with agonistic mAbs. Noteworthy, TNF secretion was not affected or regulated with these mAbs in an opposite manner (data not shown). To specifically address the role of CD11b in the pgLPS-induced IL-10 secretion, we used ligands specific for CD11b: inter-cellular adhesion molecule 1 (ICAM-1) and fibrinogen (FG) or its antagonists: blocking mAb (clone Vim12) and *N*-acetylglucosamine (GlcNAc). After such a pretreatment, cells were stimulated with pgLPS, and cytokine production was determined. Appropriate isotype controls were also applied, and we did not find their effect on cytokine secretion. Figure 7b shows that although costimulation of CD11b with ICAM-1 or fibrinogen was without the effect, IL-10 production was partially inhibited after blocking of receptor with mAbs or GlcNAc.

### Effects of Membrane-Disrupting Agent on TLR2-Induced IL-10 Production

**Fig. 8:**
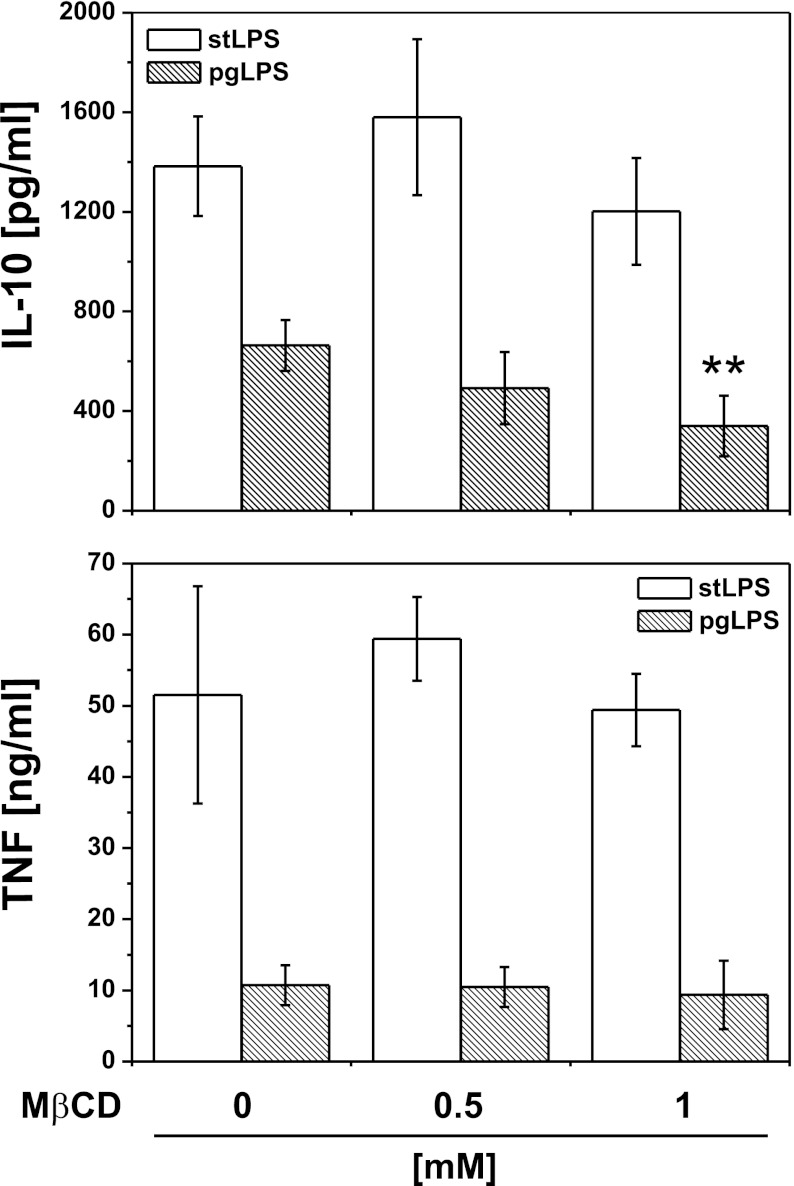
Effects of membrane-disrupting agent on TLR-induced IL-10 and TNF production. Monocytes were isolated from PBMC by adherence and placed in media supplemented with 10 % FCS. Cells were stimulated with LPS from *E. coli* (*white bars*) or *P. gingivalis* (*dashed bars*) in the presence of methyl-β-cyclodextrin (MβCD) at concentrations indicated in the figure. Supernatants were collected 20 h after stimulation, and IL-10 and TNF concentrations were determined by ELISA. Values are the mean ± SD from three independent experiments. **p < 0.01 *versus* cells cultured in the absence of MβCD and stimulated with the same type of LPS.

It has been reported that effective cell activation by PAMPs require cooperation of TLRs with several host receptors within membrane microdomains known as lipid rafts [32], which might be disrupted by oxidized PLs contributing to suppression of TLR signaling [33]. To test the possibility that effective stimulation of IL-10 and TNF secretion in varying degrees depends on lipid rafts function, we determined the effect of well-established raft-disrupting agent—MβCD—on LPS-induced cytokine production. Firstly, we carefully studied cytotoxic activity of MβCD (data not shown), and in further experiments, we used its sublethal concentrations. Figure 8 shows that whereas pgLPS-induced TNF production was lipid raft-independent, IL-10 secretion was significantly diminished by MβCD. At the same concentrations of MβCD, we did not observe the effect on TLR4-dependent cytokine production.

## DISCUSSION

In this paper, we describe an unexpected aspect of pathogen-accelerated atherosclerosis resulting from altered recognition of selected PAMPs by monocytes exposed to proatherogenic oxidized low-density lipoproteins. The dysfunctional recognition was apparently shifting the physiological balance between pro- and anti-inflammatory cytokines, represented in our study by the most biologically active members of both groups, TNF and IL-10, respectively. Strong inhibition of IL-10 induced with TLR2 and TLR4 ligands was opposed by only moderate inhibition of TNF. Thus, it appears that monocytes recognizing PAMPs in the presence of established risk factor of atherosclerosis—oxLDLs—create a cytokine milieu promoting chronic inflammation. The inhibitory effect of low-density lipoproteins depended significantly on their oxidation state, and it was not attributable to oxLDLs toxicity. A potent antiatherogenic activity of IL-10 has already been shown by many groups [20–24].

Assuming the proportion between IL-10 and TNF produced in the absence of oxLDLs as physiological, we observed that oxLDLs strongly disturbed the balance between the pro- and anti-inflammatory cytokines produced by monocytes upon TLR stimulation (Fig. 2a ,c, d, and supplementary data Table 1 and Figure 1). Irrespective of TLR-ligand used to challenge the monocytes, we observed dramatic inhibition of IL-10 and a moderate change in secretion of TNF, varying from subtle stimulation to slight inhibition. In kinetics experiments, oxLDLs inhibited the anti-inflammatory response following less or more intensely pronounced pro-inflammatory reaction (Fig. 2c, d and supplementary data Figure 1). From the same experiments, we also concluded that modulation of cytokine production by oxLDLs did not result from altered kinetics of cytokine release (supplementary data Figure 1). A spectacular example of the differential regulation of TNF and IL-10 by oxLDLs was seen upon stimulation of monocytes with LPS from *P. gingivalis* (PG). In spite of strong IL-10 suppresion, oxLDLs did not inhibit TNF production, which was even slightly increased (Fig. 2a and supplementary data Table 1). PG is a primary etiological agent of human periodontal disease, but epidemiological and experimental studies support the hypothesis that chronic infection with this bacterium may be associated with pathogen-accelerated atherosclerosis [7, 34–37]. Recently, it has been demonstrated that patients with periodontal disease are at greater risk of developing vascular dysfunction [38] as well as the association between periodontal pathogens, and subclinical atherosclerosis has been revealed [39]. Some *in vitro* studies have demonstrated that endothelial cells and monocytes respond to *P. gingivalis*, pgLPS, or FimA secreting various cytokines and chemokines [40–42], but to the best of our knowledge, there are no reports concerning the effect of oxLDLs on recognition of LPS from *P. gingivalis*. We demonstrated for the first time that oxLDLs strongly suppress production of IL-10 by monocytes challenged with PG. PAMP hypothesis of atherosclerosis postulates that the occasional presence of PAMPs in the blood may promote the activation of endothelial cells and the recruitment of monocytes into the vessel wall. Based on our results, we propose that also the anti-inflammatory response of monocytes is inhibited, thus leading to chronic inflammation. In this context, the altered recognition of PAMPs by monocytes in the presence of oxLDLs may be proposed as a new, general mechanism governing pathogen-accelerated atherosclerosis. Interestingly, we have demonstrated that the differential regulation of IL-10 and TNF is specific for monocytes. In hMDMs, both cytokines were proportionally inhibited (Fig. 2b and supplementary data: Table 1). Combined with the finding by Fuhrman *et al.* [43] that oxLDLs accelerate monocyte to macrophage differentiation, our observation suggests a coherent picture of pathogenic mechanism in which a modified, proinflammatory microenvironment can be created before monocytes differentiate into macrophages—at early stages of fatty streak development or at later stages by currently recruited cells.

In this work, we used copper oxidized LDLs, which do not occur *in vivo* and are not a physiologic molecule. Like variety of minimally modified LDLs, extensively oxidized LDLs contain a myriad of bioactive compounds including oxidized phospholipids, lysophospholipids, oxysterols, oxidized fatty acids, and variably modified ApoB (reviewed in [44]). However, it is not clear which of these components is predominant *in vivo* and has the most significant pathophysiological effect. Moreover, some of the biological effects of oxLDLs are dependent on individual components, while others are due to the complex signal provided by whole oxLDL particle. For these reasons, there is no accepted “gold standard” for preparing oxidized LDLs *ex vivo*. Nevertheless, copper oxidized LDLs resemble naturally occurring oxidized LDLs as antibodies raised against Cu^2+^LDLs recognize epitopes present *in vivo* and are successfully used to detect oxLDLs in patients [45]. We observed that both copper oxidized as well as minimally modified LDL obtained by storage of LDLs at 4°C for 6 months inhibited IL-10 production by monocytes stimulated with TLR ligands, but detailed analysis of oxidized LDL compounds was beyond of the scope of this study.

The mechanism of *P. gingivalis* LPS recognition is still controversial. It activates host cells through both TLR2 and TLR4, and this may result from heterogeneity of these molecules, containing various forms of lipid A [29]. A lipoprotein from pgLPS was suggested to be a principal component for TLR2- and highly purified lipid A for TLR4-mediated cell activation [30, 31]. On the other hand, it has been shown that in human vascular endothelial cells *P. gingivalis* LPS-induced cell activation is mediated through TLR2, and it is lipid raft-dependent as well as requires the formation of receptor complex comprising of TLR2/TLR1, CD36, and CD11b/CD18 [32]. These results support hypothesis that innate immune signaling following PG challenge is cell specific [46]. We observed that TNF secretion was dependent on TLR4 and TLR2, but IL-10 production was exclusively TLR2 dependent (Fig. 6), which is supported by strong association of TLR2 expression on monocytes with IL-10 inducibility [47].

There are conflicting data on the possibility of TLR stimulation by LDLs alone. Some authors suggested that minimally modified LDLs and their components—oxidized phospholipids—may stimulate TLR-dependent signaling [33, 48–50]. It has been reported recently that copper oxidized mmLDLs induce both proinflammatory cytokines IL-1β and IL-6 as well as anti-inflammatory cytokine IL-10, in human monocytes and U937-derived macrophages, and this effect can be assigned to CD14, TLR2, and TLR4 activation [51]. On the contrary, the evidence has been presented that neither oxPAPC nor extensively oxidized LDLs are capable of stimulating TLR2 and TLR4-dependent signaling [52, 53]. The apparent contradiction may be in part explained by a suggestion that pro- or anti-inflammatory activities of oxidized LDLs may depend on their concentration [54] experimental designs and/or variable LDLs preparations [44]. Nevertheless, in our hands, spontaneously oxidized mmLDLs and oxLDLs alone did not stimulate detectable TNF or IL-10 secretion (data not shown).

It is clear that the ability of both TLR4 and TLR2 receptors to activate cells relies on “accessory proteins,” soluble serum or membrane bound factors such as CD14 or LBP [55, 56] The serum factors were also identified as key targets for oxidized phospholipids, which specifically inhibit TLR2 and TLR4 by competitive interaction [27, 28]. Consequently, we were interested if IL-10 and TNF production by monocytes upon stimulation via TLR2 and TLR4 was dependent on soluble serum molecules. As shown in Fig. 4, IL-10 and TNF production responded differently to serum concentration only following TLR2 activation. Precisely, IL-10 production was relatively low in serum-free media and at 1 % FCS and was strongly augmented in 10 % FCS, but for maximal TNF production, supplementation with 1 % FCS was sufficient and further raising the FCS concentration suppressed TNF secretion. It is noteworthy that TNF production induced with LPS from *P. gingivalis* appeared completely independent on serum (Fig. 4). These results correlate with data showing that serum-soluble CD14 effectively transferred *P. gingivalis* LPS to TLR2 plus TLR1, but poorly to TLR4 [29], and our observation of TLR2-dependent production of IL-10 by monocytes stimulated with pgLPS. Assuming that sCD14 and LBP are main targets for oxPL, the described dependence of cytokine production on serum factors correlated well with the inhibition pattern shown in Fig. 2a. To expand this observation, we treated cells with oxLDLs and then washed them out before stimulation. From this experiment, we concluded that inhibition of IL-10 production occurred mainly by competitive interaction of oxLDLs with accessory proteins as removing the preincubation medium before the stimulation abolished the inhibitory effect of lipoproteins. The “unblocking” effect of medium change was most pronounced for Pam3CSK4 and pgLPS. The apparent IL-10-promoting effect of serum attracted our attention to a possibility that some still unrecognised serum “accessory molecules” may participate the pgLPS-TLR2-IL-10 pathway. As the effect of serum was apparent only in freshly adhered monocytes but neither in hMDMs (where adherence is established) nor in elutriated monocytes incubated in polypropylene tubes (where adherence is prevented), we assumed that the serum “accessory protein” is one of its factors of adherence. Consequently, we tried to substitute FCS using purified fibronectin, fibrinogen, or vitronectin. Among the tested proteins, only vitronectin demonstrated a concentration-dependent effect on IL-10 production (Fig. 7). Previous studies concerning vitronectin receptors expression on human monocytes have produced conflicting results. Some groups suggested that, although freshly isolated monocytes lack surface expression of β3 and β5 integrins, it appeared gradually during monocyte adhesion and maturation [57]. In our hands, expression of αvβ3 on monocytes 5 h post-isolation was evident while αvβ5 was barely detactable. Still, pretreatment with vitronectin receptors agonistic mAbs (clones: RUU-PL 7F12, P1F6, or LM609), which alone did not stimulate cytokine production, resulted in dramatic increase in pgLPS-induced production of IL-10 (Fig. 7b). The agonistic effect was also observed in serum-free media (data not shown) suggesting that costimulation through TLR2 and β3 and β5 integrins leads to increased IL-10 production.

As CD11b is naturally involved in adherence of monocytes to solid substratum, we have demonstrated that some ligands or antagonists of CD11b influenced the IL-10 production induced by TLR2 ligands (Fig. 7b). Significantly, we have not seen any effect of ICAM-1 or fibrinogen, what may suggest that CD11b is involved through lateral interaction with other receptor(s) rather than by direct binding of vitronectin or other serum “accessory molecules.” As TLR2 and 4 form a functional surface complex with integrin CD11b/CD18 [32], our results may suggest that vitronectin receptor is a novel partner in this interaction. An interaction between CD11b and vitronectin has been described in neutrophils [58]. The most likely sites for such interactions are cholesterol-rich membrane platforms containing arrays of PRRs and other surface proteins, which are now proposed to play a critical role in interaction of PAMPs with immune cells [59]. Indeed, disruption of lipid rafts with methyl-β-cyclodextrin inhibited the effect of pgLPS on IL-10 production, while TNF-production was not afflicted. TLR-containing lipid rafts harbor not only other PRRs (e.g., Dectin-1 [60]) but also receptors and proteins, which have not been directly connected to pattern recognition (e.g., HSP90 [61]). We have previously reported functional interactions of CD11b with CD16, which stabilized other surface molecules (phosphatidylserine, annexin I) not previously known to interact with the integrin complex [62–64]. In this paper, we suggest that also vitronectin receptor may function as a component of TLR signaling platforms.

Vitronectin has become an important mediator in the pathogenesis of coronary atherosclerosis because of its ability to bind platelet glycoproteins and mediate platelet adhesion and aggregation at sites of vascular injury (extensively reviewed in [65, 66]). The involvement of vitronectin and CD11b in the described mechanism is also supported by interesting clinical findings: Firstly, elevated levels of vitronectin are connected with coronary atherosclerosis and are postulated to be the result of a compensatory mechanism [67]; on the other hand, inhibition of vitronectin receptor enhances the uptake of oxLDL and differentiation of monocytes/macrophages into foam cells [68]; secondly, transmigrating monocytes from patients with coronary artery disease have lower expression of CD11b [69]; thirdly, in children with hypercholesterolemia, cell surface expression of CD11b and CD18 on PBMC was significantly decreased [70]; finally, Toll-like receptor 2 and 4 stimulation produced an enhanced inflammatory response in human obese patients with atherosclerosis [71].

Summarizing, it seems justified to assume that proatherogenic oxidized LDLs disturbed the balance between pro- and anti-inflammtory cytokines produced by monocytes upon TLR2 and TLR4 stimulation. IL-10 release was strongly suppressed by oxLDLs with no respect to the TLR ligand used for activation. In contrast, TNF production was reduced to a lesser extent than IL-10, unaffected or even slightly increased, when stimulated with LPS from *P. gingivalis*, one of infectious agent of atherosclerosis. Although the precise mechanisms of observed cytokine modulation remain to be determined, our results highlight the differential dependence of IL-10 and TNF production on serum accessory molecules. Significantly, vitronectin or/and its receptor appear to be the main target of oxidized phospholipids inhibitory activity on TLR-induced activation.

## Electronic supplementary material

Below is the link to the electronic supplementary material.

Figure 1 Time course of TLR-induced IL-10 and TNF secretion by monocytes cultured in the presence of oxidized LDLs. Monocytes were isolated from PBMC by adherence and placed in media supplemented with 10 % FCS. Cells were cultured alone (*cyan squares*) or treated for 30 min with oxidized LDLs at the 15 μg/ml (*red circles*), and then stimulated with stLPS (*left panel*) or pgLPS (*right panel*). Supernatants were collected at time points indicated in the figure, and IL-10 and TNF concentrations were determined by ELISA. Values are the mean ± SD from three independent experiments. (GIF 26 kb)

High resolution image (EPS 489 kb)

Table 1 Cytokine production by (A) monocytes and (B) monocyte-derived macrophages (hMDM) in response to PAMPs in the presence (+) or absence (−) of oxLDLs. Monocytes and hMDMs placed in media supplemented with 10 % FCS were cultured alone or treated for 30 min with oxidized LDLs at the 15 μg/ml, and then stimulated with selected PAMPs. Supernatants were collected 20 h after stimulation, and IL-10 and TNF concentrations were determined by ELISA. Values are the mean ± SD from at least five independent experiments. Unstimulated cells (control or cultured in the presence of oxLDL) did not produce detectable amounts of IL-10 and TNF (data not shown). (DOC 48 kb)
