# Supplementary figures and images for: Oxidized LDLs Inhibit TLR-induced IL-10 Production by Monocytes: A New Aspect of Pathogen-Accelerated Atherosclerosis
Source: Inflammation. 2012 May 4;35(4):1567–84. doi: 10.1007/s10753-012-9472-3 (PMC3397235; doi:10.1007/s10753-012-9472-3)

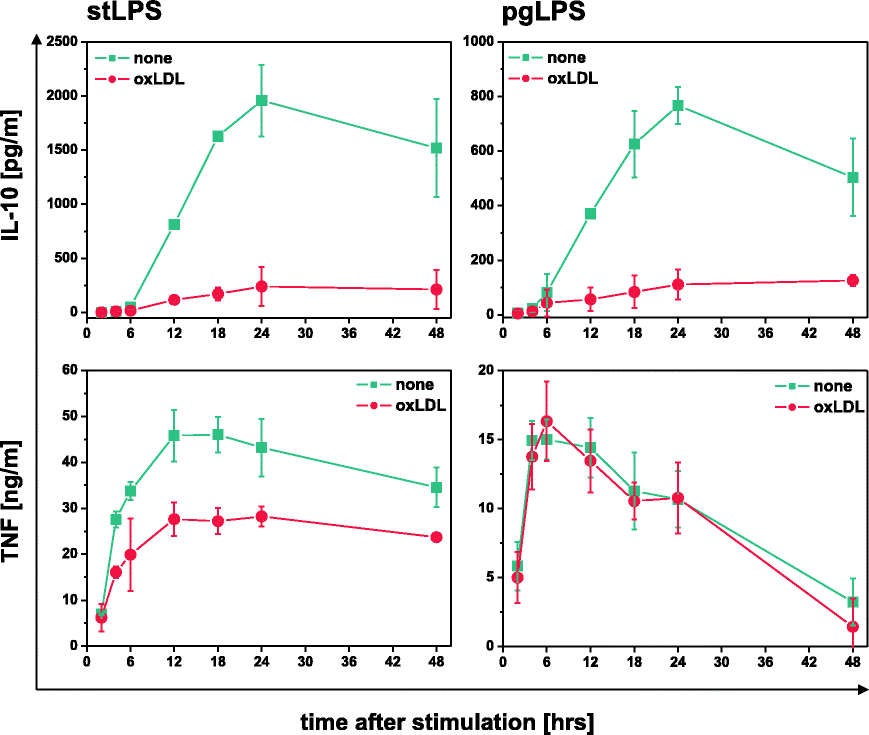

Supplement: Supplementary file 1 — Time course of TLR-induced IL-10 and TNF secretion by monocytes cultured in the presence of oxidized LDLs. Monocytes were isolated from PBMC by adherence and placed in media supplemented with 10 % FCS. Cells were cultured alone (cyan squares) or treated for 30 min with oxidized LDLs at the 15 μg/ml (red circles), and then stimulated with stLPS (left panel) or pgLPS (right panel). Supernatants were collected at time points indicated in the figure, and IL-10 and TNF concentrations were determined by ELISA. Values are the mean ± SD from three independent experiments. (GIF 26 kb) [file 10753_2012_9472_Fig9_ESM.gif]
