# Supplementary material for: Oxidized LDLs Inhibit TLR-induced IL-10 Production by Monocytes: A New Aspect of Pathogen-Accelerated Atherosclerosis
Source: Inflammation. 2012 May 4;35(4):1567–84. doi: 10.1007/s10753-012-9472-3 (PMC3397235; doi:10.1007/s10753-012-9472-3)
Supplement: Supplementary file 3 — Cytokine production by (A) monocytes and (B) monocyte-derived macrophages (hMDM) in response to PAMPs in the presence (+) or absence (−) of oxLDLs. Monocytes and hMDMs placed in media supplemented with 10 % FCS were cultured alone or treated for 30 min with oxidized LDLs at the 15 μg/ml, and then stimulated with selected PAMPs. Supernatants were collected 20 h after stimulation, and IL-10 and TNF concentrations were determined by ELISA. Values are the mean ± SD from at least five independent experiments. Unstimulated cells (control or cultured in the presence of oxLDL) did not produce detectable amounts of IL-10 and TNF (data not shown). (DOC 48 kb) [file 10753_2012_9472_MOESM2_ESM.doc]

**Table 1. Cytokine production by (A) monocytes and (B) monocyte-derived macrophages (hMDM) in response to PAMPs in the presence (+) or absence (-) of oxLDLs.** Monocytes and hMDMs placed in media supplemented with 10%FCS were cultured alone or treated for 30 min. with oxidized LDLs at the 15μg/ml, and then stimulated with selected PAMPs. Supernatants were collected 20h after stimulation and IL-10 and TNF concentrations were determined by ELISA. Values are the mean ± S.D. from at least five independent experiments. Unstimulated cells (control or cultured in the presence of oxLDL) did not produce detectable amounts of IL-10 and TNF (data not shown).

| **A.** | **stLPS** | | **upLPS** | | **pgLPS** | | **Pam2CSK4** | | **Pam3CSK4** | | **oxLDL** |
| --- | --- | --- | --- | --- | --- | --- | --- | --- | --- | --- | --- |
| **TNF** | 51,98 | ± 17,39 | 19,92 | ± 7,61 | 12,66 | ± 3,10 | 11,75 | ± 3,99 | 13,02 | ± 3,99 | - |
| **[ng/ml]** | 28,88 | ± 10,14 | 3,85 | ± 3,23 | 14,34 | ±4,27 | 9,19 | ± 7,16 | 8,28 | ± 7,20 | + |
| **IL-10** | 1815 | ± 196 | 910 | ± 213 | 696 | ± 179 | 613 | ± 123 | 635 | ± 183 | - |
| **[pg/ml]** | 447 | ± 277 | 28 | ± 37 | 121 | ± 86 | 96 | ± 68 | 87 | ± 106 | + |

| **B.** | **stLPS** | | **upLPS** | | **pgLPS** | | **Pam2CSK4** | | **Pam3CSK4** | | **oxLDL** |
| --- | --- | --- | --- | --- | --- | --- | --- | --- | --- | --- | --- |
| **TNF** | 8,61 | ± 2,65 | 3,57 | ± 1,59 | 1,07 | ± 0,34 | 0,63 | ± 0,12 | 0,52 | ± 0,09 | - |
| **[ng/ml]** | 5,28 | ± 0,26 | 0,75 | ± 0,66 | 0,74 | ± 0,39 | 0,47 | ± 0,21 | 0,24 | ± 0,18 | + |
| **IL-10** | 627 | ± 193 | 285 | ± 79 | 171 | ± 25 | 170 | ± 39 | 71 | ± 8 | - |
| **[pg/ml]** | 276 | ± 60 | 85 | ± 36 | 83 | ± 4 | 117 | ± 4 | 26 | ± 6 | + |
